# Supplementary material for: Alternatively spliced NFKB1 transcripts enriched in Andean Aymara modulate inflammation, HIF and hemoglobin
Source: Nat Commun. 2025 Feb 19;16:1766. doi: 10.1038/s41467-025-56848-0 (PMC11840074; doi:10.1038/s41467-025-56848-0)
Supplement: Supplementary file 1 — Reporting summary [file 41467_2025_56848_MOESM1_ESM.pdf]

nature portfolio

Corresponding author(s): Josef T. Prchal and Yonghee Lee

Last updated by author(s): Oct 23, 2024

Reporting Summary

Nature Portfolio wishes to improve the reproducibility of the work that we publish. This form provides structure for consistency and transparency in reporting. For further information on Nature Portfolio policies, see our Editorial Policies and the Editorial Policy Checklist.

Statistics

For all statistical analyses, confirm that the following items are present in the figure legend, table legend, main text, or Methods section.

n/a	Confirmed	
☐	☒	The exact sample size (*n*) for each experimental group/condition, given as a discrete number and unit of measurement
☐	☒	A statement on whether measurements were taken from distinct samples or whether the same sample was measured repeatedly
☐	☒	The statistical test(s) used AND whether they are one- or two-sided<br>*Only common tests should be described solely by name; describe more complex techniques in the Methods section.*
☒	☐	A description of all covariates tested
☐	☒	A description of any assumptions or corrections, such as tests of normality and adjustment for multiple comparisons
☐	☒	A full description of the statistical parameters including central tendency (e.g. means) or other basic estimates (e.g. regression coefficient) AND variation (e.g. standard deviation) or associated estimates of uncertainty (e.g. confidence intervals)
☒	☐	For null hypothesis testing, the test statistic (e.g. *F*, *t*, *r*) with confidence intervals, effect sizes, degrees of freedom and *P* value noted<br>*Give P values as exact values whenever suitable.*
☒	☐	For Bayesian analysis, information on the choice of priors and Markov chain Monte Carlo settings
☒	☐	For hierarchical and complex designs, identification of the appropriate level for tests and full reporting of outcomes
☐	☒	Estimates of effect sizes (e.g. Cohen's *d*, Pearson's *r*), indicating how they were calculated

Our web collection on statistics for biologists contains articles on many of the points above.

Software and code

Policy information about availability of computer code

Data collection	No software used.
Data analysis	Preprocessing for sequencing data: FastQC, Trimmomatic v0.32;<br>Mapping and processing for whole-genome sequencing data: hisat2 v2.1.0, Picard v2.1.1, GATK v4.1, SAMtools v1.10, and VarScan v2.3.9;<br>Mapping and processing for RNA-seq data: STAR v2.5, RSEM v1.3.0, rMATS v3.2.5, and DESeq2;<br>Gene-set over-representation analysis: ConsensusPathDB (http://cpdb.molgen.mpg.de/);<br>Protein-protein network: StringDB web tool (https://string-db.org/);<br>Analysis codes used in this study is available in GitHub (https://github.com/hangost/AymaraSplicing).

For manuscripts utilizing custom algorithms or software that are central to the research but not yet described in published literature, software must be made available to editors and reviewers. We strongly encourage code deposition in a community repository (e.g. GitHub). See the Nature Portfolio guidelines for submitting code & software for further information.

Data

Policy information about availability of data

All manuscripts must include a data availability statement. This statement should provide the following information, where applicable:

- Accession codes, unique identifiers, or web links for publicly available datasets
- A description of any restrictions on data availability
- For clinical datasets or third party data, please ensure that the statement adheres to our policy

The transcriptome data of granulocytes from Aymara and European individuals generated in this study have been deposited in the NCBI database under accession code PRJNA1204927 [https://submit.ncbi.nlm.nih.gov/subs/sra/SUB14978799/overview].

Research involving human participants, their data, or biological material

Policy information about studies with human participants or human data. See also policy information about sex, gender (identity/presentation), and sexual orientation and race, ethnicity and racism.

Reporting on sex and gender	We used samples and data from both males and females. Regarding hemoglobin levels, since hemoglobin levels differ between males and females, we included only male participants in this manuscript to reduce variation related to the menstrual cycle and pregnancies, which commonly lead to iron deficiency in young females and would introduce unrelated variables to the hemoglobin measurements.
Reporting on race, ethnicity, or other socially relevant groupings	We collected samples from Aymaras and Europeans living in La Paz, Bolivia. Ethnicity was self-reported. However, we did not categorize the individuals based on social or other socioeconomic status.
Population characteristics	Two ethnically distinct populations (Aymara and European) living in La Paz, Bolivia were studied for high altitude research. Patients with polycythemia vera and essential thrombocythemia were diagnosed by Dr. Josef T. Prchal. We did not limit the collection of samples based on age or gender.
Recruitment	Participants were recruited through Dr. Amaru and Dr. Prchal. All participants provided informed consent before participating in the study. The study protocol was reviewed and approved by the Institutional Review Board of San Andres University Medical School, La Paz, Bolivia and University of Utah, Salt Lake City, USA. There was no bias in the recruitment process. Efforts were made to ensure a diverse and representative sample, and the recruitment strategy aimed to include individuals from various backgrounds. As a result, we do not anticipate any significant self-selection or other biases that could impact the results of the study.
Ethics oversight	The study was approved by the Institutional Review Board of San Andres University Medical School, La Paz, Bolivia, and University of Utah, Salt lake City, Utah, USA.

Note that full information on the approval of the study protocol must also be provided in the manuscript.

Field-specific reporting

Please select the one below that is the best fit for your research. If you are not sure, read the appropriate sections before making your selection.

☒ Life sciences ☐ Behavioural & social sciences ☐ Ecological, evolutionary & environmental sciences

For a reference copy of the document with all sections, see nature.com/documents/nr-reporting-summary-flat.pdf

Life sciences study design

All studies must disclose on these points even when the disclosure is negative.

Sample size	For the initial analysis, we studied 10 Aymaras and 4 Europeans. Although this is a small sample size, it was enough to identify key patterns and generate initial ideas. To confirm these findings, we tested them in a larger group of 55 Aymaras and 18 Europeans. This two-step process allowed us to explore trends first and then confirm them with more data. The smaller group was used to save resources, while the larger group provided enough data to confirm meaningful differences and ensure the results were reliable. We used appropriate statistical methods, like paired, unpaired t-tests, or Mann Whitney test, to analyze the data. Even though we didn't calculate the required sample size beforehand, validating the findings in the larger group confirmed the reliability of our conclusions.
Data exclusions	We did not exclude any data.
Replication	We repeated our experiments at least three times, and the data remained consistent.
Randomization	We categorized the samples according to genotype only. There was no gender or age bias.
Blinding	We conducted a correlation study involving genotypes, phenotypes, and expression levels of AS-NFKB1. Blinding was not applicable to our study.

Reporting for specific materials, systems and methods

We require information from authors about some types of materials, experimental systems and methods used in many studies. Here, indicate whether each material, system or method listed is relevant to your study. If you are not sure if a list item applies to your research, read the appropriate section before selecting a response.

Materials & experimental systems

n/a	Involved in the study
☐	☒ Antibodies
☐	☒ Eukaryotic cell lines
☒	☐ Palaeontology and archaeology
☒	☐ Animals and other organisms
☒	☐ Clinical data
☒	☐ Dual use research of concern
☒	☐ Plants

Methods

n/a	Involved in the study
☒	☐ ChIP-seq
☒	☐ Flow cytometry
☒	☐ MRI-based neuroimaging

Antibodies

Antibodies used

rabbit anti-GFP (#ab290, Abcam), rabbit anti-NFKB1 (#13586, Cell Signaling) and rabbit anti B-actin (#ab8227, Abcam), NF-kB (Cat. NBP2-22178; Novus), GAPDH (MA5-15738-HRP, Thermo Fisher Scientific), and Histone H3 (ab1791, Abcam)

Validation

1.rabbit anti-GFP (#ab290, Abcam): This antibody was verified for western blot experiments and used in 3369 publications (https://www.abcam.com/en-us/search?facets.categoryType=Primary+Antibodies&sorting=relevance&keywords=gfp&utm_source=google&utm_medium=cpc&gad_source=1&gclid=Cj0KCQjwveK4BhD4ARIsAKy6pMKnvmR8MR41Ipk7F5ouZXIyeV1GYF_R6fjbRIeLApU3sUCv2Gc2H0caAssDEALw_wcB&gclsrc=aw.ds&productcode=AB290&view=publications)
2. rabbit anti-NFKB1 (#13586, Cell Signaling): This antibody was verified for western blot experiments and used in 141 publications (https://www.cellsignal.com/products/primary-antibodies/nf-kb1-p105-p50-d4p4d-rabbit-mab/13586?srsltid=AfmBOooEFWxX204v82rWh9dUpnImjAq5djEsVrnbHUj6kYQvhu46nxKl)
3. abbit anti B-actin (#ab8227, Abcam): This antibody was verified for western blot experiments and used in 4034 publications (https://www.abcam.com/en-us/products/primary-antibodies/beta-actin-antibody-ab8227)
4. NF-kB (Cat. NBP2-22178; Novus): This antibody was verified for western blot experiments and ony specific to human NF-kB protein used in 2 publications (https://www.novusbio.com/products/nfkb-p105-p50-antibody-5d10d11_nbp2-22178?srsltid=AfmBOoo8dnX1LXH9FmKYCeeyS5DcZkvNa7O4je6vynm3sAHMaAeQgKvy)
5. GAPDH (MA5-15738-HRP, Thermo Fisher Scientific): This antibody was verified for western blot application and used in 42 publications. (https://www.thermofisher.com/antibody/product/GAPDH-Loading-Control-Antibody-clone-GA1R-Monoclonal/MA5-15738-HRP)
6. Histone H3 (ab1791, Abcam): This antibody was verified for western blot application and used in 5010 publications. (https://www.abcam.com/en-us/products/primary-antibodies/histone-h3-antibody-nuclear-marker-and-chip-grade-ab1791).

Eukaryotic cell lines

Policy information about cell lines and Sex and Gender in Research

Cell line source(s)

HEK293 was derived from human embryonic kidney cells. HL60 cells were derived from promyeoloblasts isolated from whole blood of a patient with acute promyelocytic leukemia.

Authentication

None of cell lines used in this experiment was authenticated.

Mycoplasma contamination

All cell lines were tested negative for mycoplasma contamination.

Commonly misidentified lines
(See ICLAC register)

N/A

Plants

Seed stocks	*Report on the source of all seed stocks or other plant material used. If applicable, state the seed stock centre and catalogue number. If plant specimens were collected from the field, describe the collection location, date and sampling procedures.*
Novel plant genotypes	*Describe the methods by which all novel plant genotypes were produced. This includes those generated by transgenic approaches, gene editing, chemical/radiation-based mutagenesis and hybridization. For transgenic lines, describe the transformation method, the number of independent lines analyzed and the generation upon which experiments were performed. For gene-edited lines, describe the editor used, the endogenous sequence targeted for editing, the targeting guide RNA sequence (if applicable) and how the editor was applied.*
Authentication	*Describe any authentication procedures for each seed stock used or novel genotype generated. Describe any experiments used to assess the effect of a mutation and, where applicable, how potential secondary effects (e.g. second site T-DNA insertions, mosiacism, off-target gene editing) were examined.*
