# Supplementary material for: Alternatively spliced NFKB1 transcripts enriched in Andean Aymara modulate inflammation, HIF and hemoglobin
Source: Nat Commun. 2025 Feb 19;16:1766. doi: 10.1038/s41467-025-56848-0 (PMC11840074; doi:10.1038/s41467-025-56848-0)
Supplement: Supplementary file 2 — Supplementary Information [file 41467_2025_56848_MOESM2_ESM.pdf]

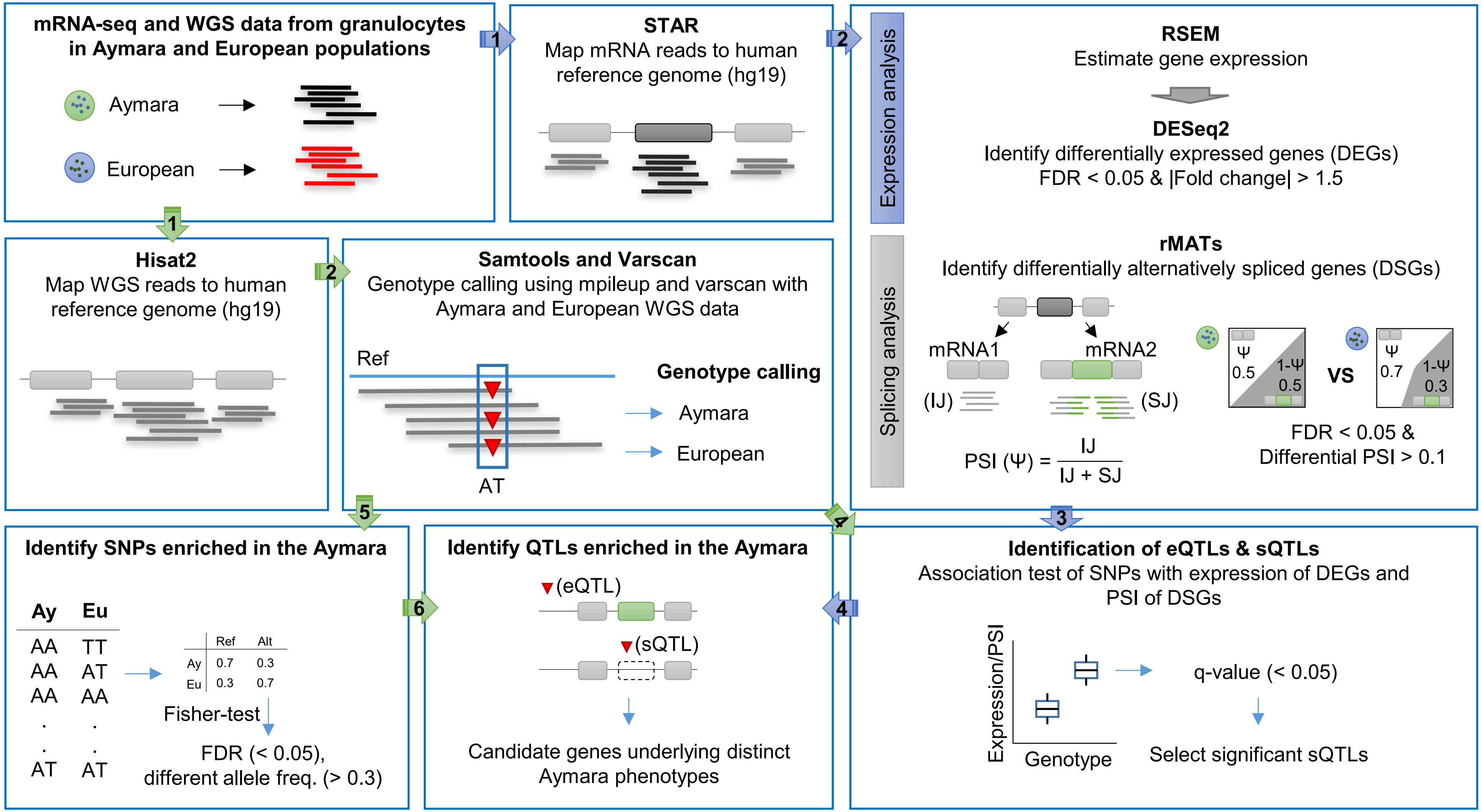

Fig. S1. Overall research design of the computational analysis. WGS: whole-genome sequencing; FDR: false discovery rate; eQTL: expression quantitative trait loci; sQTL: splicing quantitative trait loci; PSI: percent spliced in; DEG: differential expressed gene.

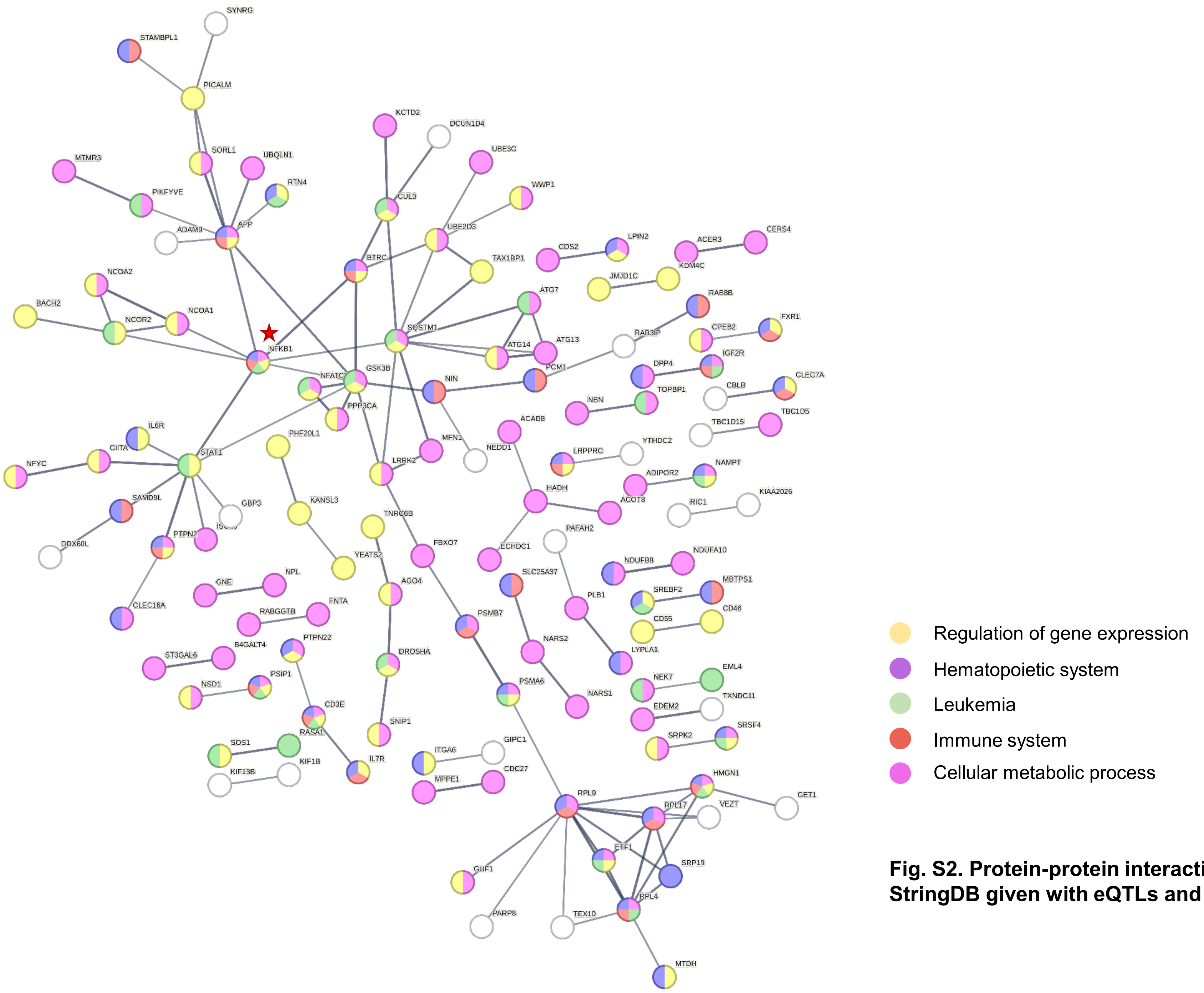

Fig. S2. Protein-protein interaction network from StringDB given with eQTLs and sQTLs genes

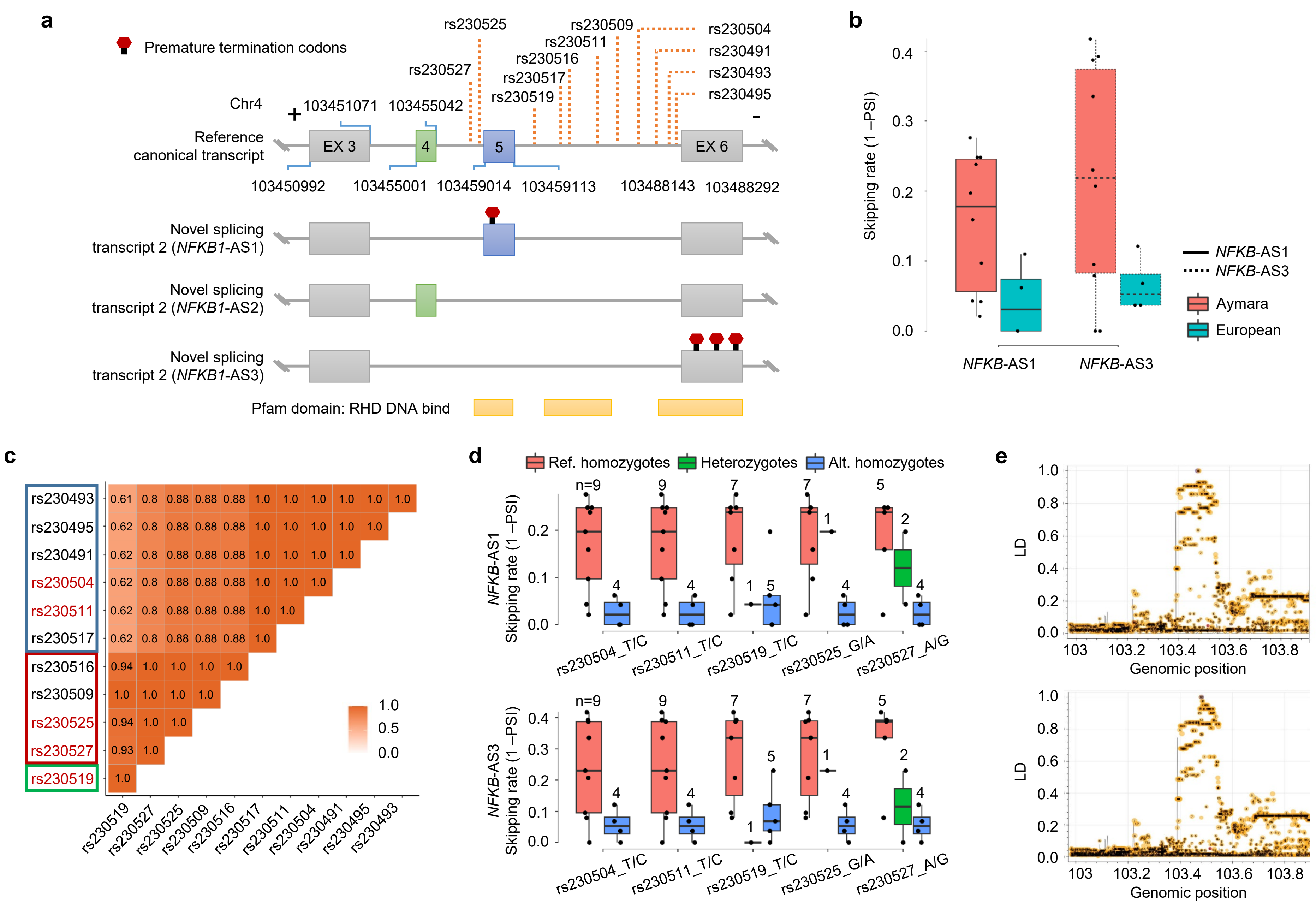

Fig. S3. Novel exon skipping of exon 4 and exon 5 in *NFKB1*. (a) A gene model of reference canonical *NFKB1* and alternative splicing (AS)-*NFKB1*, and genomic coordinates of 11 sQTLs associated with the novel AS of exon 4 and 5 in *NFKB1*. (b) Boxplot of the skipping rate (percent spliced in (PSI) - 1) distribution of *NFKB1-AS1* and *NFKB1-AS3* in Aymara and European populations. *Y*-axis and *x*-axis indicate skipping rates and AS forms of *NFKB1*, respectively; n=10 for Aymara, n=4 for European. (c) A pairwise genetic similarity heatmap of 11 sQTLs associated with the NFKB1-AS forms: The 1.0 means exactly same genotypes between two SNPs in Aymara WGS samples. (d) Boxplots of skipping rate (percent spliced in (PSI) - 1) distribution of *NFKB1-AS1* and *NFKB1-AS3* according to genotypes in each of five representative sQTLs. *Y*-axis and *x*-axis indicate skipping rates and sQTLs, respectively. The number of samples are described above boxplots. (e) Values of the linkage disequilibrium statistics R^2 between A. rs230511 and nearby SNPs, and B. rs230504 and nearby SNPs calculated in LDlink (Machiela and Chanock 2015). Each circle indicates an SNP represented in the PEL panel of the 1000 Genomes data.

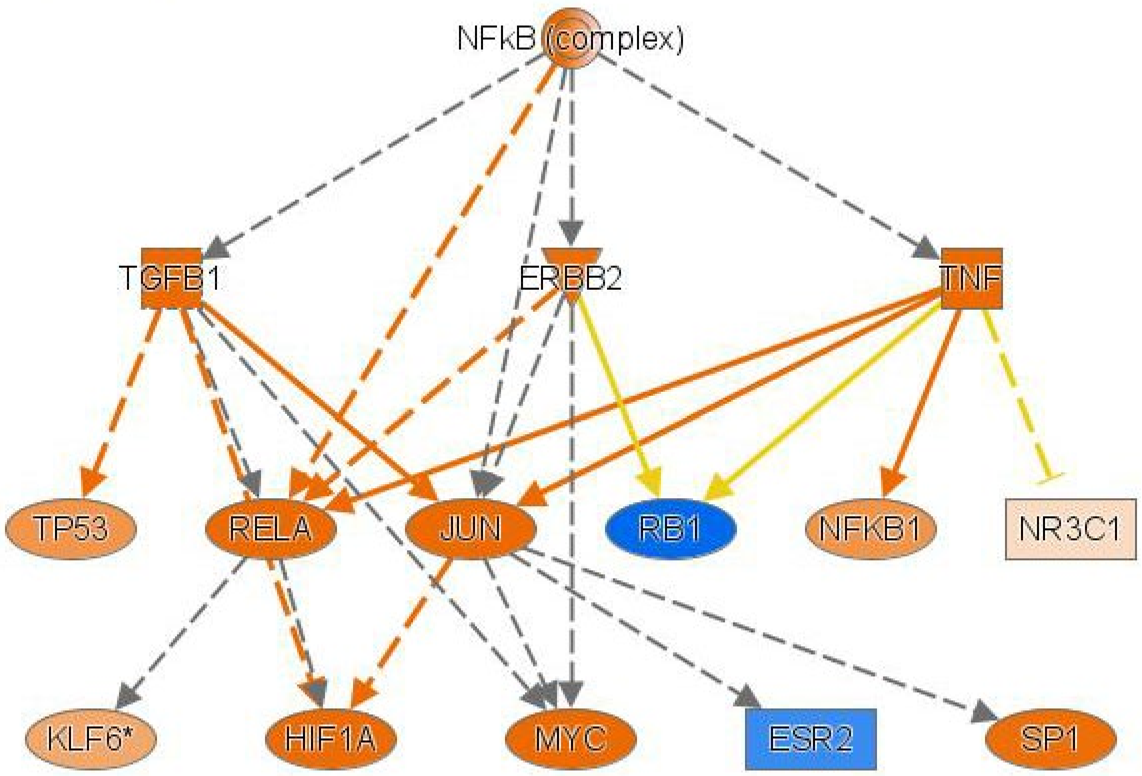

© 2000-2023 QIAGEN. All rights reserved.

Fig. S4. The NF-κB complex was predicted to be an activated upstream regulator, as determined using QIAGEN Ingenuity Pathway Analysis.
A dataset with gene identifiers and their corresponding measurement values was uploaded into the application. Each identifier was linked to its associated entity in QIAGEN's Knowledge Base. A threshold of adjusted p-value < 0.05 and fold change log2 > 1 was applied to identify molecules with significantly altered expression. These molecules, referred to as Network Eligible Molecules, were integrated into a global molecular network constructed from QIAGEN Knowledge Base data. Networks of these molecules were subsequently generated algorithmically based on their connectivity.

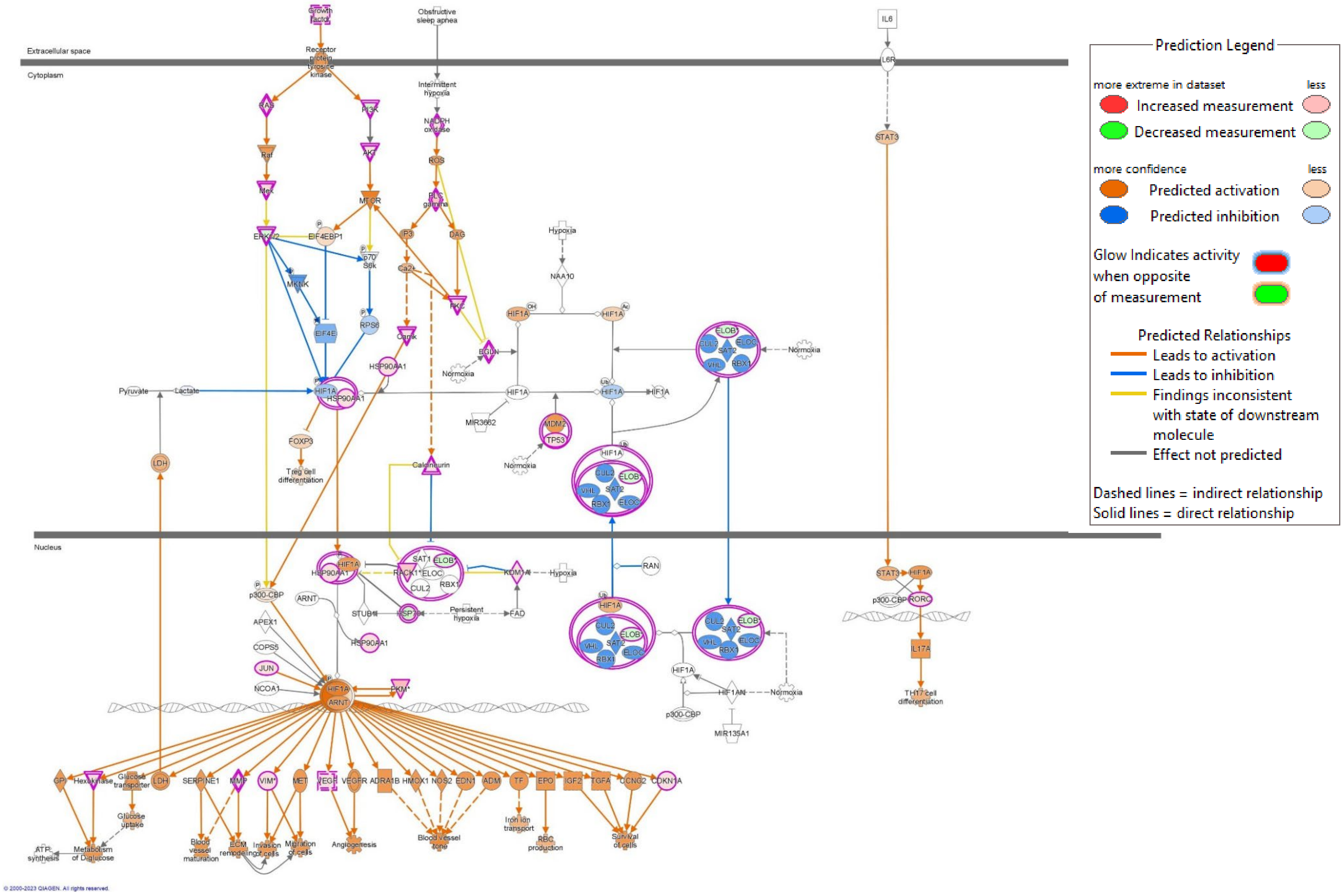

Fig. S5. HIF-1a signaling pathway was upregulated in Aymara as determined using QIAGEN Ingenuity Pathway Analysis.
Canonical pathways analysis identified the most significant pathways from the QIAGEN Ingenuity Pathway Analysis library that were relevant to the dataset. Molecules from the dataset that met the threshold of adjusted p-value < 0.05 and fold change log2 > 1, and were associated with a canonical pathway in the QIAGEN Knowledge Base, were included in the analysis.

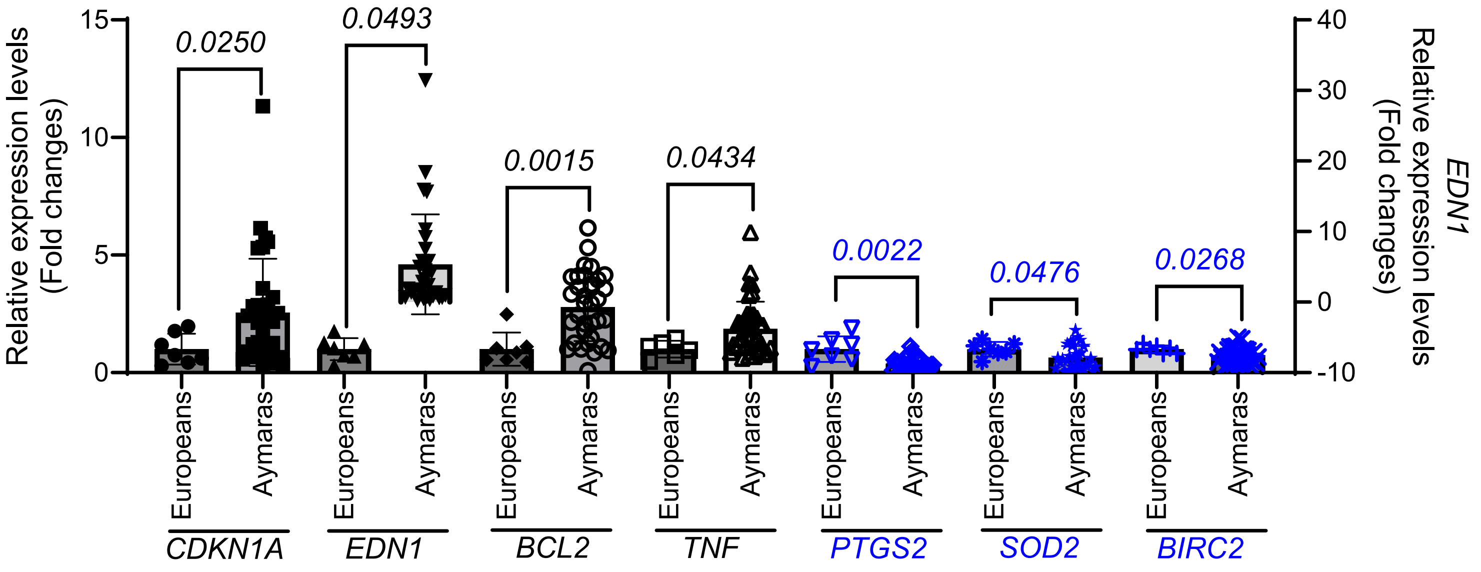

Fig. S6. Verification by qRT-PCR of expression levels of genes dysregulated in Aymara that are regulated by both NF-kB and HIF.
qRT-PCR was used to verify the findings from whole transcriptome analysis. *CDKN1A*, *EDN1*, *BCL2*, and *TNF* (in black) were upregulated while *PTGS2*, *SOD2*, and *BIRC2* (in blue) were downregulated in Aymara (n=7) compared to Europeans (n=34). Expression levels are expressed as fold change. The p-values were calculated using a two-tailed Mann-Whitney test. All data are presented as mean ± standard deviation (SD).

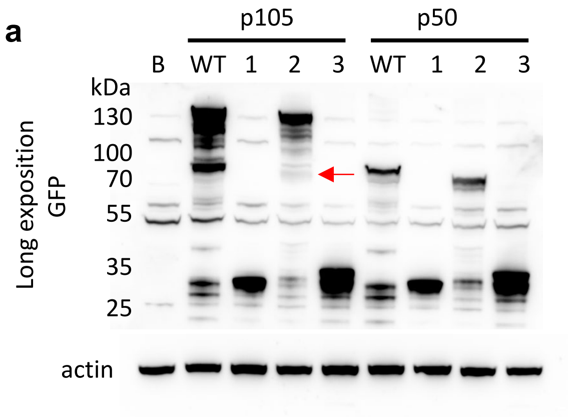

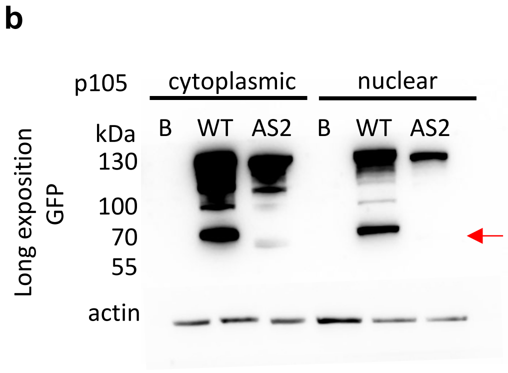

Fig. S7. Alternatively spliced *NFKB1* variants cause disrupted protein translation and processing. Long exposition for GFP antibody; please see Manuscript Fig. 6a and b for details.

Figure 7a Cytoplasmic protein

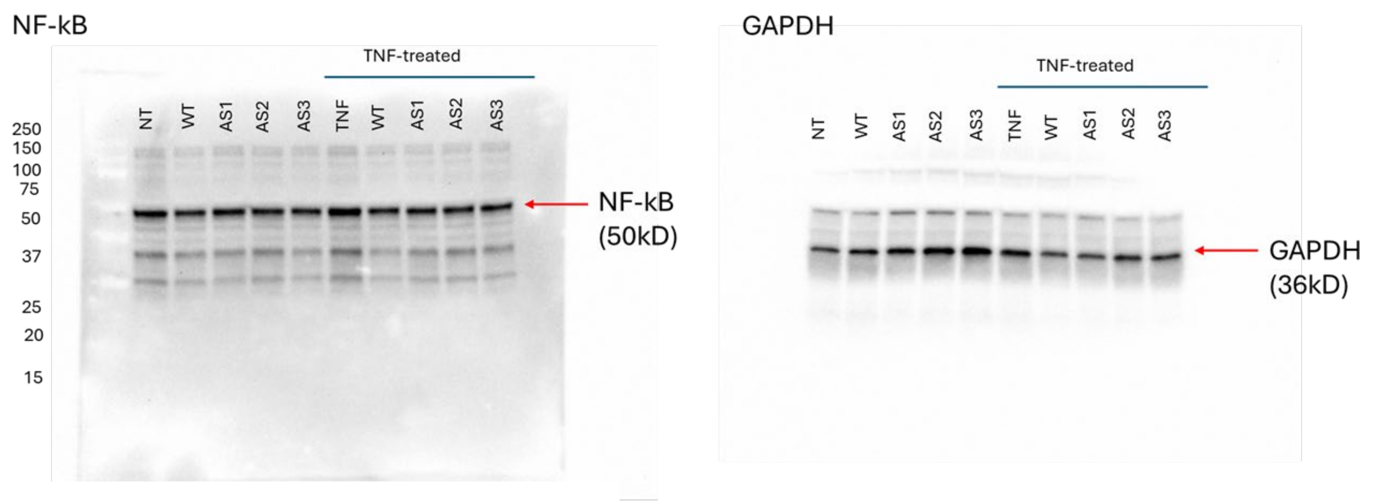

Figure 7b Nuclear protein

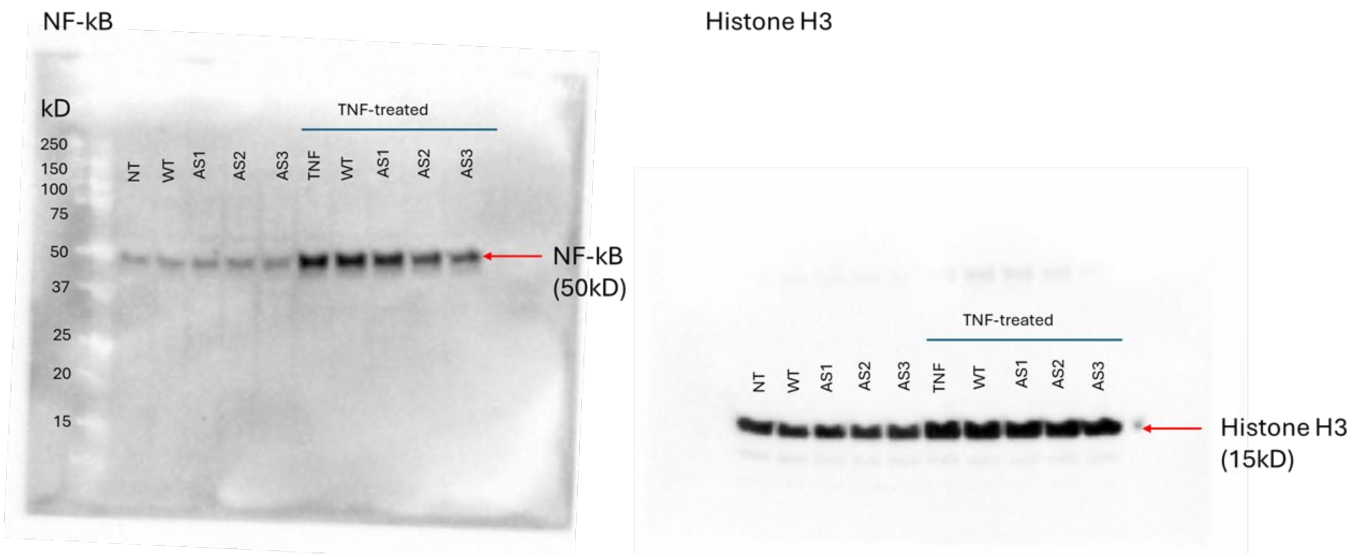

Fig. S8. Uncropped western blot image of Figure 7 (a) and (b); please see Manuscript Fig. 7 (a) and (b) for details.

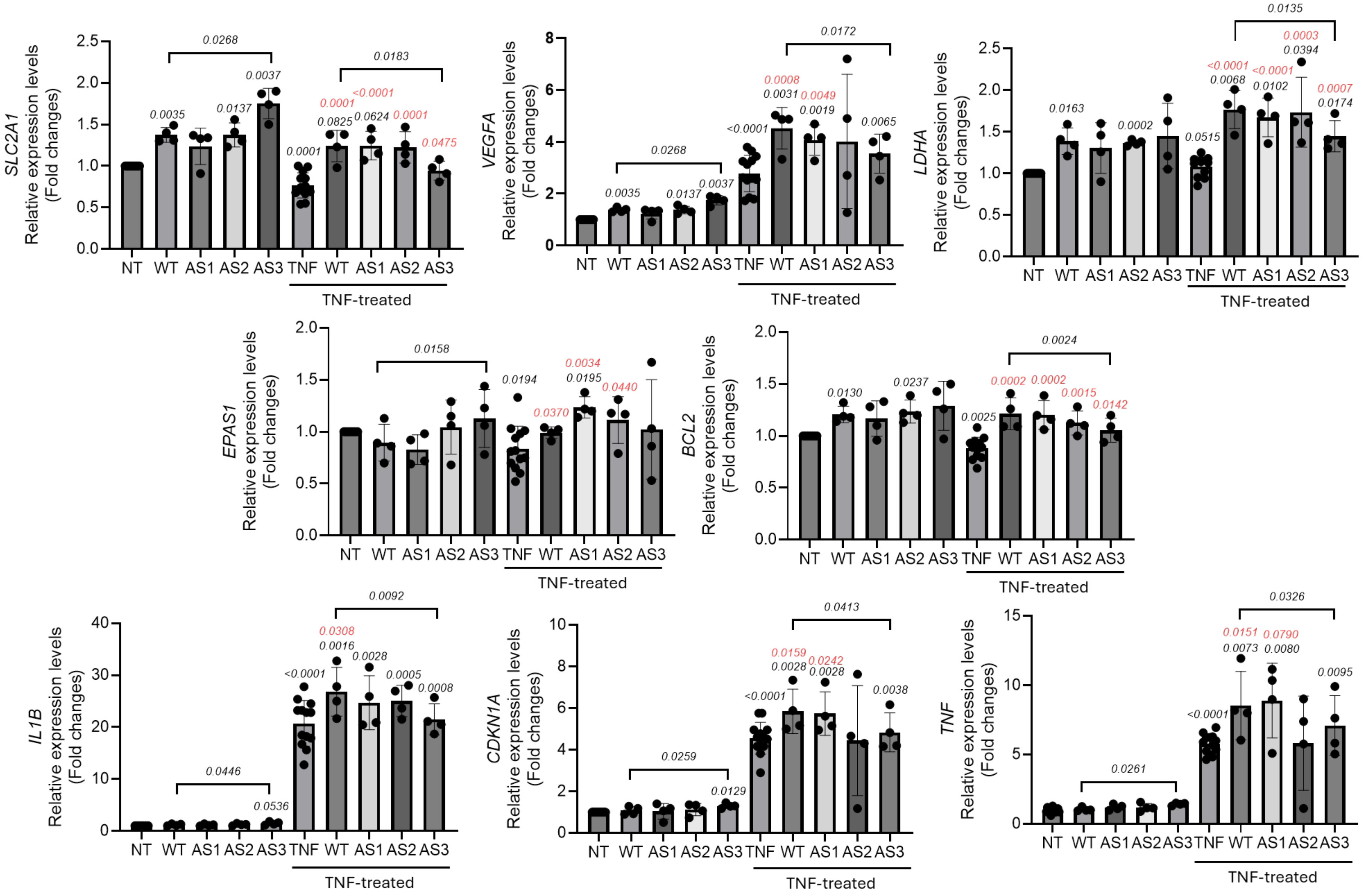

Fig. S9. HIF-targeted, inflammatory gene expression levels under AS-*NFKB1*-overexpressed and TNF treated HL60.
Transcript levels of HIF-targeted genes (*SLC2A1*, *VEGFA*, *LDHA*, *EPAS1*, and *BCL2*) and inflammatory genes (*IL1B*, *CDKN1A*, and *TNF*) were measured in three HL60 cell lines overexpressing either canonical NFKB1 (WT) or NFKB1-AS (AS1, AS2, AS3) with and without TNF treatment. NT: no-treatment; WT: canonical *NFKB1*; AS1: *NFKB1*-AS1; AS2: *NFKB1*-AS2; AS3: *NFKB1*-AS3; Black asterisks: P value compared to NT; Red asterisks: P value compared to TNF; Four independent overexpression experiments were performed under identical experimental conditions. P-values were calculated using a two-tailed paired t-test (for comparisons to NT or between WT and AS) or an unpaired t-test (for comparisons to TNF). All data are presented as mean ± standard deviation (SD).

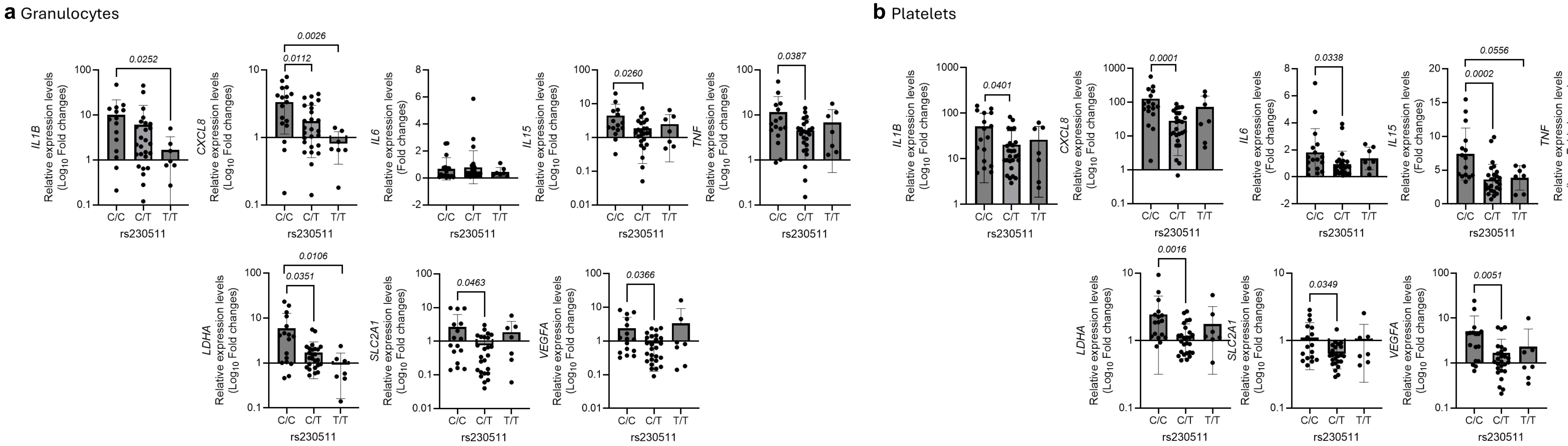

Fig. S10. Inflammatory gene and HIF-targeted gene expression levels in polycythemia vera (PV) and essential thrombocythemia (ET) and their correlation with *NFKB1* SNP rs230511.

Inflammatory genes, including *IL1B*, *CXCL8*, *IL6*, *IL15*, and *TNF* transcript levels, as well as HIF-targeted genes, including *SLC2A1*, *VEGFA*, and *LDHA* transcript levels, were measured in (a) granulocytes and (b) platelets from 34 PV and 16 ET patients (C/C = 17, C/T = 26, T/T = 7). The *P*-values were calculated using a two-tailed Mann-Whitney test. All data are presented as mean ± standard deviation (SD).
