# Supplementary material for: Alternatively spliced NFKB1 transcripts enriched in Andean Aymara modulate inflammation, HIF and hemoglobin
Source: Nat Commun. 2025 Feb 19;16:1766. doi: 10.1038/s41467-025-56848-0 (PMC11840074; doi:10.1038/s41467-025-56848-0)
Supplement: Supplementary file 3 — Description Of Additional Supplementary File [file 41467_2025_56848_MOESM3_ESM.pdf]

Description of additional supplementary files

Supplementary Data 1. List of differentially expressed genes in Aymara

Supplementary Data 2. Significant Gene Ontology and canonical pathway in differentially expressed genes

Supplementary Data 3. List of differential alternative splicing exons in Aymara

Supplementary Data 4. Significant Gene Ontology and canonical pathway in differential splicing genes

Supplementary Data 5. eQTLs associated with differentially expressed genes

Supplementary Data 6. sQTLs associated with differential alternative splicing exons

Supplementary Data 7. eQTLs and sQTLs where Aymara has different allele frequencies compared to the gnomAD European

Supplementary Data 8. Correlation of inflammatory gene expression with AS-NFKB1 and canonical NFKB1

Supplementary Data 9. Correlation of NF-kB target genes with AS-NFKB1 and canonical NFKB1

Supplementary Data 10. Correlation of HIF target genes with AS-NFKB1 and canonical NFKB1

Supplementary Data 11. Correlation of both NF-kB and HIF target genes with AS-NFKB1 and canonical NFKB1
